# Urban Flood Resilience Evaluation Based on Heterogeneous Data and Group Decision-Making

**DOI:** 10.3390/e26090755

**Published:** 2024-09-03

**Authors:** Xiang He, Yanzhu Hu, Xiaojun Yang, Song Wang, Yingjian Wang

**Affiliations:** 1 School of Intelligent Engineering and Automation, Beijing University of Posts and Telecommunications, Beijing 100876, China; hexiang6@bupt.edu.cn (X.H.); wongsangwongsang@163.com (S.W.); wangyingjian@bupt.edu.cn (Y.W.); 2 Unit 63892 of PLA, Luoyang 471000, China; yangxiaojun2007@gmail.com

**Keywords:** urban flood resilience, group decision-making, heterogeneous data, indicator system, normal cloud model

## Abstract

In recent years, urban floods have occurred frequently in China. Therefore, there is an urgent need to strengthen urban flood resilience. This paper proposed a hybrid multi-criteria group decision-making method to assess urban flood resilience based on heterogeneous data, group decision-making methodologies, the pressure-state–response model, and social–economic–natural complex ecosystem theory (PSR-SENCE model). A qualitative and quantitative indicator system is formulated using the PSR-SENCE model. Additionally, a new weighting method for indicators, called the synthesis weighting-group analytic hierarchy process (SW-GAHP), is proposed by considering both intrapersonal consistency and interpersonal consistency of decision-makers. Furthermore, an extensional group decision-making technology (EGDMT) based on heterogeneous data is proposed to evaluate qualitative indicators. The flexible parameterized mapping function (FPMF) is introduced for the evaluation of quantitative indicators. The normal cloud model is employed to handle various uncertainties associated with heterogeneous data. The evaluations for Beijing from 2017 to 2021 reveal a consistent annual improvement in urban flood resilience, with a 14.1% increase. Subsequently, optimization recommendations are presented not only for favorable indicators such as regional economic status, drainability, and public transportation service capacity but also for unfavorable indicators like flood risk and population density. This provides a theoretical foundation and a guide for making decisions about the improvement of urban flood resilience. Finally, our proposed method shows superiority and robustness through comparative and sensitivity analyses.

## 1. Introduction

Since the onset of the Industrial Revolution, there has been a significant increase in humanity’s impact on the natural environment. This has resulted in climate change and environmental degradation, posing a threat to the delicate global ecological balance and the survival of mankind. The UN Office for Disaster Risk Reduction (UNDRR) released the Global Assessment Report (GAR 2023), which emphasizes how critical it is to build resilience to face and overcome adversity. Almost 3000 natural disasters occurred worldwide between 2021 and 2023. Of these, meteorological disasters constituted the majority, accounting for over 60%. The relentless recurrence of events like heavy rains, floods, hurricanes, and tornadoes has resulted in significant loss of life, extensive property damage, and societal instability.

Scholars have given the idea of resilient cities much attention. In 2013, the Urban Resilience Framework was unveiled by the Rockefeller Foundation as part of the “100 Resilient Cities” initiative, which chose 100 communities globally for practical application and study [1]. As a result, the concept of “urban flood resilience” was created. Currently, urban flood resilience has become a subject of extensive research. Orense et al. [2] developed a coastal community resilience evaluation system from a risk perspective, applying the analytic hierarchy process (AHP) and Delphi technique for vulnerability assessment and management. Kotzee et al. [3] introduced a socioecological index and applied it to three flood-prone cities in South Africa, choosing 24 social and ecological factors to evaluate the regional distribution of flood impacts. Dong and his colleagues [4] analyzed flood control strategies for cattle farms in Heilongjiang Province using 15 factors to characterize the natural environment, culture, community, and economy. 

Although previous studies have greatly advanced the assessment of urban flood resilience, these studies only considered the ability to withstand flood disasters in urban flood resilience. As a result, the identification of weak points in the resilience process and a greater comprehension of the theories and mechanisms behind urban resilience are overlooked. Additionally, many evaluation methods predominantly focus on qualitative discussions, with only a few combining both subjective and objective elements. 

In light of these considerations, this research endeavors to analyze the resilience process and its characteristics. To identify relevant factors, a conceptual framework known as the PSR-SENCE model is first built. Subsequently, to determine the primary contributing factors, a thorough systematic review (SR) was performed. Finally, the ultimate influencing factors are determined through group decision-making involving experienced experts in relevant fields. This represents the paper’s first major contribution.

In research on methods for evaluating urban flood resilience, some studies tend to construct specific indicators for evaluating disaster resilience [3,5,6]. However, urban flood resilience is a multidimensional, comprehensive cross-temporal capability, and the scope of consideration of a single indicator is too one-sided and difficult to fully express its characteristics. Multi-Criteria Decision-Making (MCDM) is an extension of decision theory. Hence, the MCDM method, which serves as a means to assess or rank objects based on a range of diverse criteria, has found extensive application in flood disaster assessment. However, in the research on calculating indicator weights via GAHP, most previous papers focused on aggregating individual preferences to group consensus [7,8,9,10] but ignored the importance of determining the weights of decision-makers. This paper proposes a synthesis weights GAHP (SW-GAHP) method, which comprehensively considers decision-makers’ intrapersonal consistency and interpersonal consistency to determine the decision-makers’ weights. This constitutes the second primary contribution of this paper.

In previous multi-criteria evaluation approaches [11,12,13,14,15], there has often been a focus on assessing quantitative indicators, with limited discussion on qualitative indicators. The integration of both quantitative and qualitative evaluation methods has been constrained, resulting in evaluation outcomes lacking a deeper understanding and failing to comprehensively capture the overall problem. This paper adopts an approach that combines both quantitative and qualitative evaluations. In terms of assessing quantitative indicators, traditional methods [11,12,13,14,15] typically employed the linear normalization method. However, some indicators do not adhere to linear patterns. Thus, this paper employs the flexible parameterized mapping function (FPMF) for the evaluation of quantitative indicators. The FPMF can accommodate both linear and nonlinear change patterns, making it more versatile. This forms the third primary contribution of this paper.

In terms of assessing qualitative indicators, group decision-making (GDM) technology is currently used extensively. However, previous expert empowerment methods for GDM treatment, such as direct customized allocation by a super decision-maker, are usually subjective. Yang et al. [16] adopted an objective weighting approach that combines the degree of uncertainty and the consistency of participants’ evaluation results based on normal cloud models. However, their consistency calculation only considered the expected values of the evaluation results. The fourth primary contribution of this paper lies in describing the distance computation technique of the normal cloud model and introducing a novel technique for group decision-making known as the extensional group decision-making technology (EGDMT).

Numerous uncertainties exist in both quantitative and qualitative indicators, and researchers have employed various concepts to characterize these uncertainties. Li et al. [17,18] presented the idea of a cloud model that both represents randomness and fuzziness based on probability theory and type-2 fuzzy sets. Recently, Yang et al. [16] and numerous other scholars [19,20,21,22] have explored decision-making methodologies grounded in cloud models. NCMs stand out by being capable of jointly modeling fuzziness and randomness while offering more straightforward and intuitive operations. Consequently, the use of NCMs has grown significantly in recent years. The introduction of a heterogeneous decision-making information fusion method is the paper’s fifth important contribution. This method can combine exact numbers, statistical data, interval numbers, linguistic terms, NCMs, and linguistic expressions.

To summarize, this paper’s primary contributions are as follows:

(1). A conceptual framework for evaluating urban flood resilience was established by the PSR-SENCE model, and an extensive SR approach was integrated to produce an indicator system for thorough evaluation.
(2). A novel indicator weight determination method named SW-GAHP is proposed, which takes into account both intrapersonal and interpersonal consistency while considering the decision-making quality of different decision-makers.
(3). The use of the FPMF method for resilience calculation of quantitative indicators can handle both linear and nonlinear patterns for indicators with different meanings, thereby accurately reflecting their actual impact on urban flood resilience.
(4). An EGDMT method is proposed for evaluating qualitative indicators, where decision-maker weights are determined based on the uncertainty degree and group consensus bias of their decision-making information.
(5). A heterogeneous decision-making information fusion method is introduced for evaluating qualitative indicators.

The remainder of this paper is organized as follows: Section 2 provides the prerequisite knowledge needed for understanding the paper. Section 3 outlines the construction of the evaluation indicator system. Section 4 introduces the urban flood resilience evaluation methodology, including the SW-GAHP, FPMF, and EGDMT methods. In Section 5, the proposed method is applied to a case study of Beijing’s flood resilience evaluation, and recommendations are made in light of the findings’ analysis. Section 6 offers a comprehensive and detailed comparison with previous methods. Finally, Section 7 offers concluding thoughts.

## 2. Preliminaries

### 2.1. Cloud Model Theory

Let T be a qualitative term that is defined on the discourse universe U={u}. Let x∈U be a random instance of T, and $\mu_{T}(x)\in [0,1]$ represents the degree of certainty that x belongs to *T*, which corresponds to a stochastic variable exhibiting stable trends. The cloud model describes a concept through three numerical characteristics: expectation (*Ex*), entropy (*En*), and hyper-entropy (*He*).

*Ex* represents the mathematical average of the cloud droplet’s position within the universe. It serves as the most representative point embodying the qualitative concept and acts as the sample that is most commonly used to quantify the concept.

En measures the degree of uncertainty, taking into account the concept’s fuzziness and randomness. On one hand, En quantifies the randomness of the qualitative concept, reflecting the dispersion of cloud droplets representing the concept. On the other hand, En measures the ambivalence of the qualitative concept, indicating the range of acceptable cloud droplet values within the universe space.

He is the uncertainty degree of En.

#### 2.1.1. Normal Cloud Model

The normal cloud model (NCM) is based on the normal distribution and Gauss membership function [17].

**Definition** **1** ([23])**.** *Let U be the universe of discourse, and let $\tilde{A}$ be a qualitative concept in U. If x∈U is a random instantiation of concept $\tilde{A}$, which satisfies $x∼N\left(Ex,E{n^{\prime }}^{2}\right)$, $En^{\prime }∼N\left(En,He^{2}\right)$, and the certainty degree of x belonging to concept $\tilde{A}$ satisfies*

(1) $$y=e^{-\frac{{\left(x-Ex\right)}^{2}}{2{\left(En^{\prime }\right)}^{2}}}$$

Therefore, a normal cloud is the distribution of *x* throughout the universe *U*.

#### 2.1.2. Operation Rules

Within the same universe, the arithmetic operation rules [24,25,26,27,28] for NCMs $C_{1}=\left(Ex_{1},En_{1},He_{1}\right)\;\mathrm{and}\;C_{2}=\left(Ex_{2},En_{2},He_{2}\right)$ are defined as follows:

(2) $$C_{1}+C_{2}=\left(Ex_{1}+Ex_{2},\sqrt{En_{1}^{2}+En_{2}^{2}},\sqrt{He_{1}^{2}+He_{2}^{2}}\right)$$

(3) $$C_{1}-C_{2}=\left(Ex_{1}-Ex_{2},\sqrt{En_{1}^{2}+En_{2}^{2}},\sqrt{He_{1}^{2}+He_{2}^{2}}\right)$$

(4) $$C_{1}\times C_{2}=\left(Ex_{1}Ex_{2},\sqrt{{\left(En_{1}Ex_{2}\right)}^{2}+{\left(En_{2}Ex_{1}\right)}^{2}},\sqrt{{\left(He_{1}Ex_{2}\right)}^{2}+{\left(He_{2}Ex_{1}\right)}^{2}}\right)$$

(5) $$T_{1}/T_{2}=\left(\frac{Ex_{1}}{Ex_{2}},\sqrt{{\left(\frac{En_{1}}{Ex_{2}}\right)}^{2}+{\left(\frac{Ex_{1}En_{2}}{Ex_{2}^{2}}\right)}^{2}},\sqrt{{\left(\frac{He_{1}}{Ex_{2}}\right)}^{2}+{\left(\frac{Ex_{1}He_{2}}{Ex_{2}^{2}}\right)}^{2}}\right)$$

**Definition** **2.** *Let $C_{1}=\left(Ex_{1},En_{1},He_{1}\right)$ and $C_{2}=\left(Ex_{2},En_{2},He_{2}\right)$ be two NCMs in U. The distance between $C_{1}$ and $C_{2}$ is defined as follows:*

(6) $$\mathrm{bias}(C^{d},\bar{C})=\sqrt{\varepsilon {\left|Ex^{d}-\bar{E}x\right|}^{2}+\phi {\left|En^{d}-\bar{E}n\right|}^{2}+\varphi {\left|He^{d}-\bar{H}e\right|}^{2}}.$$

*where ε,ϕ,φ are three proportionality coefficients, 0<ε<1,0<ϕ<1,0<φ<1.*

**Definition** **3** ([24])**.** *Let $C_{i}=\left(Ex_{i},En_{i},He_{i}\right)$ (i=1,2,…,n) be a set of NCMs in U. The synthetic operator is a mapping CS: $C^{n}\to C$.*

(7) $$CS\left(C_{1},C_{2},\ldots ,C_{n}\right)=\left(\frac{1}{n}\sum_{i=1}^{n}Ex_{i},\frac{1}{6}\left(\underset{i}{\max}\left(Ex_{i}+3En_{i}\right)-\underset{j}{\min}\left(Ex_{j}\left.\left.\left.-3En_{j}\right)\right),\sqrt{\sum_{i=1}^{n}He_{i}^{2}}\right)\right.\right.\right.$$

The *En* and *He* of the synthetic NCM are both larger than or equivalent to those of each individual NCM. Consequently, the synthetic NCM includes a wider range of uncertainties, thereby providing a broader and more generalized coverage of information.

**Definition** **4** ([24,26])**.** *Let $C_{i}=\left(Ex_{i},\;En_{i},\;He_{i}\right)(i=1,\;2,\;\ldots ,\;n)$ be a set of NCMs in U. A mapping CWA: $C^{n}\to C$, serves as the weighted average operator following*

(8) $$CWA\left(C_{1},\;C_{2},\;\ldots ,\;C_{n}\right)=\sum_{i=1}^{n}w_{i}C_{i}/\sum_{i=1}^{n}w_{i}$$

*where $w_{i}$ is the weight of $C_{i}$.*

According to the inference [24,26], if $w_{i}\in [0,1],\;i=1,2,\ldots ,n$ is a real number and $\sum_{i=1}^{n}w_{i}=1$; then, Equation (8) is easy to understand as follows:

(9) $$CWA\left(C_{1},C_{2},\ldots ,C_{n}\right)=\left(\sum_{i=1}^{n}w_{i}Ex_{i},\sqrt{\sum_{i=1}^{n}{\left(w_{i}En_{i}\right)}^{2}},\sqrt{\sum_{i=1}^{n}{\left(w_{i}He_{i}\right)}^{2}}\right)$$

### 2.2. Conversion of Heterogeneous Data

Within the framework of group decision-making, participants frequently provide assessment outcomes as heterogeneous decision information (HDI). HDI mainly appears in two variants: numeric and linguistic. The numeric form encompasses precise numbers, interval numbers, and statistical data. Conversely, linguistic form consists of linguistic terms and linguistic expressions. 

The conversion method for heterogeneous data, as combined from Yang et al. [29] and Yang et al. [16], is as follows:

#### 2.2.1. Conversion of Numeric Type Data

For an exact number, both *En* and *He* values are set to 0.

(10) v→T(v,0,0)

Let an interval number be represented as *I =* [*I^L^*, *I^U^*]. The conversion formula is as follows:

(11) $$I\to T\left(\frac{I^{L}+I^{U}}{2},\frac{I^{U}-I^{L}}{6},0\right)$$

If statistical numbers follow a normal distribution or closely approximate it, the first step is to compute the mean *µ* and standard deviation *σ* of the numbers. The statistical numbers can then be represented using an NCM as follows:

(12) $$S_{∼N(\mu ,\sigma )}\to T(\mu ,\sigma ,0)$$

#### 2.2.2. Conversion of Linguistic Type Data

When experts offer their evaluation opinions, they may tend to prefer selecting linguistic-type information because it aligns with human language conventions. Linguistic type information consists of two primary forms: linguistic terms and linguistic expressions.

**Figure 1:**
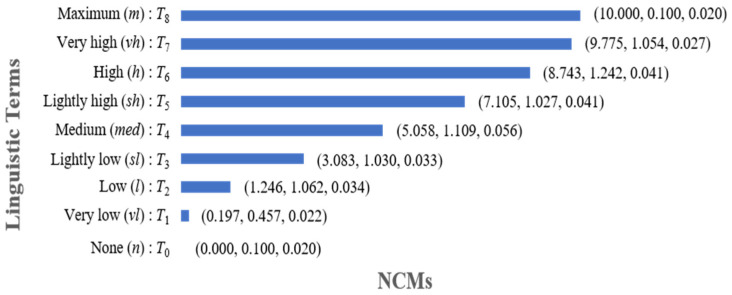
NCM-encoded nine linguistic terms.

With a dataset gathered from 175 individuals, ranging from 0 to 10 on a scale, Yang et al. converted 32 language terms into NCMs using membership function fitting and fuzzy statistics [30]. In a subsequent study [29], five of the original 32 models and two adaptive linguistic terms, along with two linguistic terms, were utilized for credibility evaluations. NCMs encoded nine linguistic terms, as shown in Figure 1 [16]. The nine linguistic terms, each associated with distinct grades as delineated in Figure 1, are also used in this study’s context.

When experts provide their evaluation opinions, they can directly choose appropriate linguistic terms from Figure 1.

Experts frequently turn to linguistic expressions to explain their opinions when faced with uncertainty while expressing hesitant opinions. However, it is important to note that linguistic expressions are not readily amenable to quantitative calculations and require conversion into hesitant linguistic term sets through specific conversion functions. Considering that Context-Free Grammar’s ($G_{H}$) definition of the comparative linguistic approach closely resembles human expression, this paper adopts the rules outlined in Definition 5 for deriving linguistic expressions.

**Definition** **5** ([31])**.** *Let $G_{H}$ be a context-free grammar, and $H=\left\{T_{0},\ldots ,T_{n}\right\}$ be a linguistic term set. The elements of $G_{H}=\left(V_{N},V_{T},I,P\right)$ are defined as follows:*

$V_{N}=\{<$*primary term*>,<*composite term*>,<*unary relation*>,<*binary relation*>*,* <*conjunction*>};

$V_{T}=\left\{lower\;than,\;greater\;than,\;at\;least,\;at\;most,\;between,\;and,\;T_{0},T_{1},\ldots ,\right.\left.T_{n}\right\};\;I\in V_{N};$

P={I::=< *primary term* >∣< *composite term* >*,*<*primary term*$>::=T_{0}\left|T_{1}\right|\ldots |T_{n}$*,* <*composite term*>::=<*unary relation*><*primary term*>∣<*binary relation* ><*primary term*><*conjunction*><*primary term*>*,* <*unary relation*>::= *lower than*∣*greater than*∣*at least*∣*at most,* <*binary relation*>::=*between,* <*conjunction*>::=*and*}.

Using the defined principles $f_{G_{H}}$, linguistic expressions are converted into hesitant cloud linguistic term sets (HCLTSs).

(13) $$\left\{\begin{array}{l} f_{G_{H}}(T_{i})=\{T_{i}|T_{i}\in H\}\\ f_{G_{H}}(\mathrm{at}\;\mathrm{most}\;T_{i})=\{T_{j}|T_{j}\in H\;\mathrm{and}\;T_{j}\leq T_{i}\}\\ f_{G_{H}}(\mathrm{lower}\;\mathrm{than}\;T_{i})=\{T_{j}|T_{j}\in H\;\mathrm{and}\;T_{j}<T_{i}\}\\ f_{G_{H}}(\mathrm{at}\;\mathrm{least}\;T_{i})=\{T_{j}|T_{j}\in H\;\mathrm{and}\;T_{j}\geq T_{i}\}\\ f_{G_{H}}(\mathrm{greater}\;\mathrm{than}\;T_{i})=\{T_{j}|T_{j}\in H\;\mathrm{and}\;T_{j}>T_{i}\}\\ f_{G_{H}}(\mathrm{between}\;T_{i}\;\mathrm{and}\;T_{j})=\{T_{k}|T_{k}\in H\;\mathrm{and}\;T_{i}\leq T_{k}\leq T_{j}\} \end{array}\right.$$

Finally, the synthetic operator given in Definition 3 is used to convert the HCLTS into an NCM.

## 3. Urban Flood Resilience Factors

### 3.1. Conceptual Framework

Rapport and Friend [32] first proposed the PSR model in 1979; subsequently, this model was altered by the United Nations Environment Programme (UNEP) and the Organization for Economic Cooperation and Development (OECD) and used to assess environmental concerns such as greenhouse gas impacts, pollution, and climate change (OECD, 2013) [33]. Three types of indicator layers make up the PSR model: pressure (P), state (S), and response (R). 

The complex ecosystem of a city is thought to be represented by the social–economic–natural complex ecosystem (SENCE). On the basis of complex ecosystem theory, Wang and Ma (1984) [34] proposed the SENCE model in the 1980s. Three distinct systems are thought to comprise cities: society, economy, and nature [35]. In 2023, Zhu and Li established the PSR-SENCE model conceptual framework. Using this model as a conceptual framework for studying urban flood resilience’s primary factors and how they interact, they identified 24 factors within three different dimensions [36]. 

Based on the PSR-SENCE model, in conjunction with the disaster system theory (DST), the framework for the concept of urban flood resilience is established within the following three dimensions: (1) Pressure Dimension: This dimension primarily quantifies the level of risk that the urban system faces in relation to urban flooding. It specifically addresses the inherent pressures placed on the composite urban ecosystem. It also includes factors contributing to flooding as outlined in DST. (2) State Dimension: This dimension primarily concerns the stable condition of the complex urban socio-economic and natural ecological system during flood disasters. It represents the environment’s capacity to bear and endure floods in the DST. It embodies urban flood resilience attributes like robustness, redundancy, and timeliness, demonstrating the city system’s ability to withstand and counteract the pressure exerted by flood disasters. Under the combined influence of flood disaster pressure and subsequent responses, the state also undergoes continuous changes. (3) Response Dimension: This dimension mainly focuses on the ability of urban entities, particularly those that bear the brunt of flood disasters, to implement response and recovery measures for urban social, economic, and natural systems post-flood disasters. It reflects the strategic, contemplative, and comprehensive qualities of urban flood resilience. Additionally, it highlights how urban institutions, such as businesses, social groups, governments, and citizens, are able to respond to flood disasters and draw lessons from both positive and negative experiences.

### 3.2. Identified Factors

Based on the aforementioned urban flood resilience conceptual framework, a comprehensive SR method is employed to identify the key factors that impact urban flood resilience. The following procedure outlines the factors that are needed to assess urban flood resilience.

(1). Screening: To begin, a search was carried out in the Web of Science Core Collection using keywords such as “urban flood resilience” or “city flood resilience” combined with “assessment” or “evaluation”. This process yielded 1172 relevant papers. Subsequently, the titles and abstracts were screened, and 96 papers remained. Finally, a full-text review was conducted, papers that were less relevant to the scope of this study were eliminated, and a total of 87 papers were obtained.
(2). Expanding: After reviewing the selected papers, relevant and high-quality references were identified, leading to an expansion in the number of references to a total of 106.
(3). Extract: Key influencing factors were extracted from the papers, and those mentioned more than once were considered. In total, 48 factors were identified and categorized according to the urban flood resilience conceptual framework.
(4). Determine: Experts with over five years of experience in the fields of urban resilience, disaster risk reduction, and emergency management were consulted. Through expert discussions and deliberations, a final indicator system comprising 23 key influencing factors was established for the evaluation of urban flood resilience.

### 3.3. Urban Flood Resilience Evaluation Indicators

**Figure 2:**
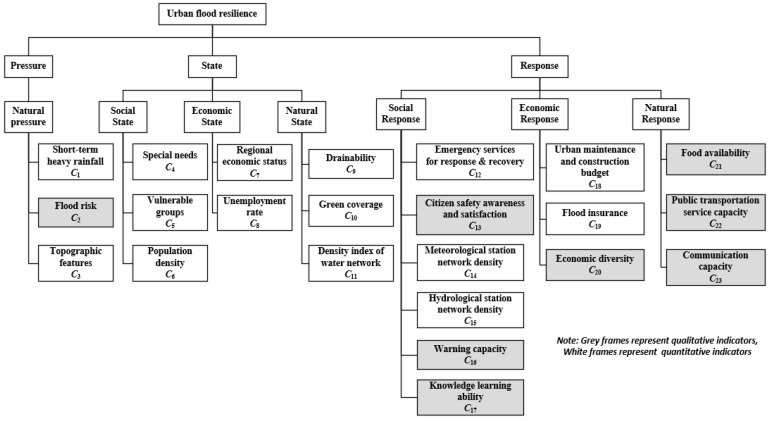
Urban flood resilience evaluation indicator system.

Following the outlined conceptual framework and factors identified, an urban flood resilience evaluation indicator system was developed, as depicted in Figure 2. 

Each indicator’s significance and assessment methodology are elaborately explained in Table 1, including a total of fifteen quantitative indicators and eight qualitative indicators.

## 4. Urban Flood Resilience Evaluation Methodology

### 4.1. Evaluation Framework

**Figure 3:**
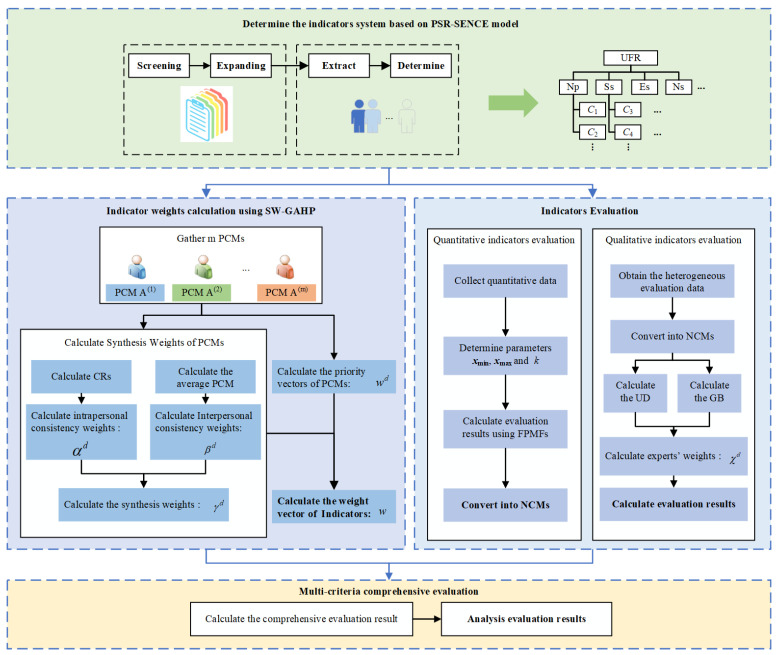
Flowchart of the methodology.

The proposed method has four primary steps, as described in Figure 3. First, an urban flood resilience evaluation indicator system is constructed (Section 3). Second, the weights of these indicators are determined using the SW-GAHP method (Section 4.2). Third, methods for evaluating quantitative and qualitative indicators are proposed (Section 4.3). Ultimately, all indicators’ evaluation results are combined using the weighted average operator for NCMs, as described in Section 4.4.

### 4.2. Indicator Weight Calculation Using SW-GAHP

The AHP is recognized as one of the most prevalent methodologies for calculating indicator weights. Additionally, GAHP models are employed for issues involving multiple participants. This section introduces an enhanced GAHP method termed SW-GAHP for the weighting of indicators.

#### 4.2.1. Synthesis Weighting Method (SWM) for DMs

Within GAHP research, the majority of previous studies have concentrated on the methods for aggregating individual preferences into group consensus [7,8,9]. However, the importance of ascertaining the weights of decision-makers (DMs) has not been adequately addressed. 

This paper introduces a novel weighting approach of DMs termed SWM, which accounts for both intrapersonal and interpersonal consistencies in Pairwise Comparison Matrices (PCMs). Intrapersonal consistency pertains to the “inconsistency level” within a single PCM. A diminished “inconsistency level” indicates fewer internal contradictions within a PCM, suggesting superior decision-making quality. Conversely, interpersonal consistency is concerned with the divergence between individual preferences and the collective consensus. A smaller “deviation” reflects a greater alignment between an individual decision-maker and the rest of the DMs.

##### Intrapersonal Consistency

Let $C=\{c_{1},c_{2},\ldots ,c_{m}\}$ represent a finite set of *m* indications, with the *i*-th indicator represented by $c_{i}$. A finite set of *k* DMs is denoted by $D=\{DM_{1},DM_{2},\ldots ,DM_{k}\}$. The m×m matrix $A^{d}=\left[a_{ij}^{d}\right],d=1,2,\ldots ,k$ represents the $PCM^{d}$ of $DM_{d}$. if $\left(a_{ij}^{d}>0\right.$ is positive for ∀i and *j*) and reciprocal $\left(a_{ji}^{d}=1/a_{ij}^{d}\right.$ for ∀i and j). Its general element $a_{ij}$ shows how many times item i is more important than element j. A PCM is said to be consistent if and only if $a_{ie}=a_{ij}a_{je}$ for ∀i,j,e,1≤i,j,e≤n. In practical decision-making problems, a PCM is likely to exhibit inconsistency. Nonetheless, the extent of consistency can vary significantly, quantifiable by the Consistency Ratio (*CR*).

(14) $$CR=\frac{CI}{RI}$$

where *RI* represents the average *CI* value of randomly created PCMs of the same size and *CI* represents *A*’s consistency index.

(15) $$CI=\frac{\lambda_{\max}-m}{m-1}$$

where $\lambda_{\max}\geq m$ is the largest eigenvalue of the PCM. In AHP, an acceptable degree of consistency is generally indicated by CR<0.1.

The “inconsistency level” of PCM provided by an individual is measured using the *CR*.

(16) $$\alpha^{d}=\frac{\max\left(0.1-CR^{d},0\right)}{\sum_{d=1}^{k}\max\left(0.1-CR^{d},0\right)}$$

where $CR^{d}$ is the *CR* of the *d-*th DM’s PCM and $\alpha^{d}$ is the weight assigned to the *d-*th DM according to intrapersonal consistency.

##### Interpersonal Consistency

Interpersonal consistency is assessed by the variance of an individual DM’s PCM from the mean of all DMs’ PCMs. This variance is determined using the Euclidean distance.

Firstly, calculate the average matrix $\bar{A}={\left[{\bar{a}}_{ij}\right]}_{m\times m}$ of all DMs as follows:

(17) $${\bar{a}}_{ij}=\frac{1}{k}(a_{ij}^{1}+a_{ij}^{2}+\ldots +a_{ij}^{k})$$

Next, calculate the deviation of an individual DM’s matrix $A^{d}$ from the average matrix $\bar{A}$.

(18) $$L_{A^{d}\bar{A}}=\sqrt{\sum_{i=1}^{m}\sum_{j=1}^{m}{\left(a_{ij}^{d}-{\bar{a}}_{ij}\right)}^{2}}$$

Lastly, as the “deviation” measure is of a cost type, its normalization and standardization methods are defined as follows:

(19) $$\begin{array}{l} L_{A^{d}\bar{A}}^{s}=1-\frac{L_{A^{d}\bar{A}}}{\sum_{d=1}^{k}L_{A^{d}\bar{A}}}.\\ \beta^{d}=\frac{L_{A^{d}\bar{A}}^{s}}{\sum_{d=1}^{k}L_{A^{d}\bar{A}}^{s}} \end{array}$$

where $\beta^{d}$ is the weight assigned to the *d-*th DM according to interpersonal consistency.

##### Synthesis Weights of DMs

By synthesizing interpersonal consistency and intrapersonal consistency, the synthesis weight of the *d-*th DM is calculated.

(20) $$\gamma^{d}=\nu \alpha^{d}+(1-\nu )\beta^{d}$$

where ν is the adjustable parameter and 0<ν<1, $\gamma^{d}$ is the synthesis weight assigned to the *d-*th DM.

#### 4.2.2. Indicator Weighting

The weight vector $DM_{d}$, represented as $w^{d}=[w_{1}^{d},w_{2}^{d},\ldots ,w_{m}^{d}]$, is the normalized eigenvector of the matrix $A^{d}$ that corresponds to the biggest eigenvalue $\lambda_{\max}^{d}$. Subsequently, the weight of indicator *C_i_*, denoted as *w_i_*, is calculated using the arithmetic weighted average of the weight vectors from *k* DMs.

(21) $$w_{i}=\sum_{d=1}^{k}\gamma^{d}w_{i}^{d},i=1,2,\cdots ,m.$$

Finally, the weight vector of *m* indicators using GAHP is ***w*** = [*w*_1_, *w*_2_..., *w_m_*].

Algorithm 1 describes the SW-GAHP algorithm.
**Algorithm 1.** SW-GAHP algorithm.**Input:** PCMs {(***A****^d^*)*_m_*_×*m*_} on *m* indicators from *k* DMs (*d* = 1, 2, …, *k*).
**Output**: ***w*** = {*w*_1_, *w*_2_, …, *w_m_*}.
**Procedure:**
 1: ***for*** *d* = 1: *k*
 2:  Calculate the largest eigenvalue $\lambda_{\max}^{d}$ and its eigenvector ***w****^d^*.
 3:  Calculate *CR^d^* by Equations (14) and (15).
 4:  Calculate each DM’s intrapersonal consistency weight $\alpha^{d}$ by Equation (16).
 5: ***end***
 6: Calculate the average matrix $\bar{A}$ of all DMs by arithmetic mean value.
 7: ***for*** *d* = 1: *k*
 8:  $\mathrm{Calculate}\;\mathrm{the}\;\mathrm{deviation}\;L_{A^{d}\bar{A}}$ of the *d*-th DM’s PCM from $\bar{A}$ by Equation (18).
 9:  Calculate each DM’s interpersonal consistency weight $\beta^{d}$ by Equation (19).
 10:  Calculate each DM’s synthesis weight $\gamma^{d}$ by Equation (20).
 11: ***end***
 12: ***for*** *i* = 1: *m*
 13:  Calculate the weight *w_i_* of indicator *C_i_* by Equation (21).
 14: ***end***

PCMs are used to determine the weight vectors of indications under the same parent indicator at each level, in line with the hierarchical structure of the indicator system. As a result, the weights of the indicators at lower levels are determined by multiplying the weight vectors in a sequential manner, starting at the highest level and working down to the lowest.

### 4.3. Indicator Evaluation

#### 4.3.1. Quantitative Indicator Evaluation Using FPMF

The quantitative data include various dimensions and trends. Traditional normalization methods often utilize linear transformations, such as the 0–1 method. This paper adopts the FPMF [71] for normalizing all quantitative indicators. By integrating three adjustable pre-defined parameters: minimum value, maximum value, and exponent *k*, which cater to both linear and nonlinear function mappings with increasing or decreasing trends, this method demonstrates superior flexibility and adaptability.

In terms of directional trends, quantitative indicators are divided into benefit-type or cost-type categories. Benefit-type indicators, where higher values are more desirable, are mapped using increasing functions. Conversely, cost-type indicators, where lower values are preferred, are mapped using decreasing functions.

Within the category of benefit-type indicators, they are further classified into convex and concave increasing functions. In the case of cost-type indicators, decreasing functions are applied, which include both convex and concave decreasing functions.

The formula for the convex increasing function (CvIF) is as follows:

(22) $$f(x)=\left\{\begin{array}{c} 0,\;x\leq x_{\min}\\ 10\cdot {\left(\frac{x-x_{\min}}{x_{\max}-x_{\min}}\right)}^{k},\;x_{\min}<x<x_{\max}\\ 10,\;x\geq x_{\max} \end{array}\right.$$

The formula for the concave increasing function (CcIF) is as follows:

(23) $$f(x)=\left\{\begin{array}{c} 10,\;x\leq x_{\min}\\ 10\cdot \left(1-{\left(\frac{x_{\max}-x}{x_{\max}-x_{\min}}\right)}^{k}\right),\;x_{\min}<x<x_{\max}\\ 0,\;x\geq x_{\max} \end{array}\right.$$

The convex decreasing function (CvDF) is represented by the following formula:

(24) $$f(x)=\left\{\begin{array}{c} 10,\;x\leq x_{\min}\\ 10\cdot {\left(\frac{x_{\max}-x}{x_{\max}-x_{\min}}\right)}^{k},\;x_{\min}<x<x_{\max}\\ 0,\;x\geq x_{\max} \end{array}\right.$$

The formula for the concave decreasing function (CcDF) is as follows:

(25) $$f(x)=\left\{\begin{array}{c} 0,\;x\leq x_{\min}\\ 10\cdot \left(1-{\left(\frac{x-x_{\min}}{x_{\max}-x_{\min}}\right)}^{k}\right),\;x_{\min}<x<x_{\max}\\ 10,\;x\geq x_{\max} \end{array}\right.$$

In this context, *x*_min_ denotes the minimum value of the quantitative indicator, while *x*_max_ represents its maximum value, and *k* (*k* ≥ 1) is an exponent that signifies varying rates of increase or decrease.

When *k* = 1, the mapping function *f*(*x*) exhibits a linear increase/decrease within the range [*x*_min_, *x*_max_]. Conversely, for values of *k* other than 1, the mapping function *f*(*x*) portrays a nonlinear increase/decrease within the same range [*x*_min_, *x*_max_].

This paper includes fifteen quantitative indicators, of which eight are increasing and seven are decreasing; only *C*_1_ is a statistical number, while the others are exact numbers. The extreme values of *C*_1_, *C*_3_, *C*_6_, *C*_7_, and *C*_8_ are selected according to the relevant level classification standards. For example, if the daily rainfall is within the range of 0–10 mm, the rainfall level is categorized as light rain. A rainfall level greater than 250 mm is classified as exceptionally heavy. Therefore, the *x*_min_ for short-term heavy rainfall *C*_1_ is set to 0 mm, and the *x*_max_ is set to 250 mm. If the elevation difference is between 0 and 200 m, it is a plain, and if it is greater than 2000 m, it is a mountain. Therefore, the *x*_min_ of topographic feature *C*_3_ is set to 0, and the *x*_max_ is 2000. When the population density is less than 1 person/km^2^, the area is an extremely sparsely populated area; when the population density is greater than 100 people/km^2^, the area is a densely populated area. Therefore, *x*_min_ of population density *C*_6_ is 0, *x*_max_ is 100, and so on. The determination of extreme values for the remaining indicators is achieved through expert consultation. Details regarding the FPMFs and associated parameters for various indicators are provided in Table 2.

By employing the FPMFs and parameters outlined in Table 2, the evaluation result $e_{i}$ of the quantitative indicator *C_i_* is determined. Subsequently, this result is transformed into an NCM $Ce_{i}$ according to the method described in Section 2.2.

#### 4.3.2. Qualitative Indicator Evaluation Using Group Decision-Making Techniques with Heterogeneous Data

Experts may represent their evaluation opinions of qualitative indicators by exact numbers, interval numbers, linguistic terms, or linguistic expressions. An exact number or interval number can be converted into an NCM by (10) or (11) in Section 2.2.1. A linguistic term can be encoded by an NCM, as shown in Figure 1 in Section 2.2.2. A linguistic expression can be represented by the context-free grammar GH and then converted into an NCM as described in Section 2.2.2. Consequently, our qualitative indicator evaluation method is capable of managing heterogeneous data, and uncertainty is modeled and propagated by NCMs.

In order to more accurately determine the weight of DMs, this paper defined the distance between two NCMs by incorporating *En* and *He* in the calculation of consistency. Thus, an EGDMT method is proposed based on the uncertainty degree and group consensus bias, which is considered more comprehensively without increasing the burden on the DMs.

(1). Uncertainness degree

The uncertainty degree (UD) of an NCM is described as follows:

(26) $$\begin{array}{c} \eta^{d}=En^{d}+3He^{d},d=1,2,\ldots ,k.\\ UD^{d}=\frac{1-\eta^{d}/\sum_{d=1}^{k}\eta^{d}}{\sum_{d=1}^{k}\left(1-\eta^{d}/\sum_{d=1}^{k}\eta^{d}\right)} \end{array}$$

where $\left(Ex^{d},En^{d},He^{d}\right)$ represents the evaluation result of the *d*-th DM, and $UD^{d}$ denotes the UD of the *d*-th DM.

(2). Group consensus bias

Group consensus bias (GB) is defined as the deviation of an individual’s evaluation result from the collective outcome. In this study, the three parameters of NCM, specifically *Ex*, *En*, and *He*, are utilized to calculate GB. 

Initially, the mean of all DMs’ evaluation results is calculated as follows:

(27) $$\bar{C}=(\bar{E}x,\bar{E}n,\bar{H}e)=\frac{1}{k}\sum_{d=1}^{k}C^{d}$$

where $C^{d}$ represents the evaluation result given by the *d*-th DM.

Subsequently, the bias of the *d*-th DM is computed as follows:

(28) $$\mathrm{bias}(C^{d},\bar{C})=\sqrt{\varepsilon {\left|Ex^{d}-\bar{E}x\right|}^{2}+\phi {\left|En^{d}-\bar{E}n\right|}^{2}+\varphi {\left|He^{d}-\bar{H}e\right|}^{2}}$$

where ε,ϕ,φ are three adjustable parameters.

Finally, the metric termed “deviation degree” is categorized as a cost-type indicator. The standardization and normalization methods for this metric are described as follows:

(29) $$\begin{array}{l} \delta^{d}=1-\frac{d(C^{d},\bar{C})}{\sum_{d=1}^{k}d(C^{d},\bar{C})},d=1,2,\cdots ,k.\\ GB^{d}=\frac{\delta^{d}}{\sum_{d=1}^{k}\delta^{d}} \end{array}$$

where $GB^{d}$ represents the weight assigned to the *d*-th DM based on the GB.

(3). Qualitative indicator evaluation

In the end, the relative weight of each DM is determined by combining the UD and GB.

(30) $$\chi^{d}=\mu UD^{d}+(1-\mu )GB^{d}$$

where μ is the adjustable parameter and 0<μ<1, and $\chi^{d}$ is the relative weight of the *d*-th DM.

Using the weighted arithmetic mean method, the evaluation result of the qualitative indicator *C_i_*, denoted as C*e_i_*, is calculated.

(31) $$Ce_{i}=\sum_{d=1}^{k}\chi^{d}e_{i}^{d},i=1,\;2,\;\cdots ,\;m$$

where $e_{i}^{d}$ (*d* = 1, 2, …, *k*) is the evaluation result of *C_i_* from *DM_d_*.

Algorithm 2 describes the EDGMT algorithm for evaluating qualitative indicators.
**Algorithm 2.** EGDMT with heterogeneous data for the evaluation of qualitative indicators.**Input:** heterogeneous data on a qualitative indicator from *k* DMs. 
**Output:** the evaluation result *Ce_i_*_._

**Procedure:**

 1: Covert heterogeneous data into NCMs (*Ex^d^*, *En^d^*, *He^d^*), (*d* = 1, 2, …, *k*). 
 2: ***for*** *d* = 1: *k*
 3:  Calculate each DM’s $UD^{d}$ by Equation (26). 
 4: ***end***
 5: Calculate average NCM $\bar{C}$ of NCMs by Equation (27).
 6: ***for*** *d* = 1: *k*
 7:  $\mathrm{Calculate}\;\mathrm{the}\;\mathrm{bias}\;d(C^{d},\bar{C})$ of an individual DM’s NCM from the average NCM of DMs by Equation (28).
 8:  Calculate each DM’s $GB^{d}$ by Equation (29).
 9:  Calculate each DM’s weight $\chi^{d}$ by Equation (30).
 10: ***end***
 11: Calculate the evaluation result of a qualitative indicator *C_i_* represented as *Ce_i_* by Equation (31).

### 4.4. Multi-Criteria Comprehensive Evaluation

The indicators’ evaluation results, both quantitative and qualitative, are $Ce=[Ce_{1},\;Ce_{2},\;\ldots ,\;Ce_{m}]$, and the weights of the indicators are $w=[w_{1},\;w_{2},\;\ldots ,\;w_{m}]$. Then, the weighted average operator of the NCM (Section 2.1.2) is used to aggregate the evaluation results of multiple indicators.

(32) $$Re=\sum_{i=1}^{m}w_{i}Ce_{i}$$

## 5. Case Study

In this section, to illustrate the implementation specifics and prove the viability of the suggested approach, a real-world example of an urban flood resilience evaluation of Beijing from 2017 to 2021 is provided.

### 5.1. Calculate the Weights of the Indicators

PCMs are required for three indications in the first level of the Beijing urban flood resilience indicator system: pressure, state, and response. As shown in Table 3, six 3 × 3 PCMs were collected from six DMs.

The six PCMs’ weight vectors were computed as follows: [0.3889, 0.2778, 0.3333], [0.3810, 0.2857, 0.3333], [0.4836, 0.1677, 0.3487], [0.3889, 0.2778, 0.3333], [0.3636, 0.3030, 0.3333], and [0.3810, 0.2857, 0.3333]. The CRs were computed as follows: 0, 0, 0.0009, 0, 0, or 0. The $L_{A^{d}\bar{A}}$ values were calculated as 0.2408, 0, 0.3253, 1.6306, 0.2408, 0.5069, and 0.3253. Then, for λ=0.5, the weights of the six DMs were calculated as 0.1761, 0.1735, 0.1329, 0.1761, 0.1679, and 0.1735. Lastly, using the weighted arithmetic mean of the six weight vectors, the weight vectors of the three indicators were calculated to be [0.3945, 0.2701, 0.3354].

Likewise, three 3 × 3 PCMs for the second-level indications in the “state” category were acquired from three DMs. It was determined that the weight vectors for the three indicators under “state” were [0.2790, 0.3208, 0.4001]. Additionally, five 3 × 3 PCMs were gathered from five DMs in order to support the “response” second-level indicators. The three indicators under “response” have been determined to have the following weight vector: [0.3922, 0.2760, 0.3318]. The weight vector for the sole indicator under ‘pressure’ was assigned a value of 1.

Furthermore, in the third-level indicator system, there are seven indicators. These seven indicators’ weight vectors were computed as follows: [0.4965, 0.3086, 0.1949], [0.1595, 0.2426, 0.5979], [0.7073, 0.2927], [0.4129, 0.2133, 0.3738], [0.1210, 0.0986, 0.2878, 0.2959, 0.1081, 0.0886], [0.5086, 0.1868, 0.3047], and [0.2327, 0.3511, 0.4122].

Consequently, the 23 bottom-level indicators’ weights were determined and are shown in Table 4. The indicator bearing the highest weight is *C*_7_, whereas *C*_4_ possesses the lowest weight. The calculated weights for the indicators were applied to all years from 2017 to 2021.

### 5.2. Obtain the Evaluation Data

The evaluation method is proposed in Section 4.3. We illustrate the process of calculating the evaluation results of indicators using data from 2021. The same process can be applied in other years.

Quantitative data from 2017 to 2021 were sourced from various authorities, including the National Statistical Yearbook, Beijing Statistical Yearbook, Chinese meteorological stations, China Water Resources News, and the Beijing Water Authority. These data include 15 indicators: *C*_1_, *C*_3_ to *C*_12_, *C*_14_, *C*_15_, *C*_18_, and *C*_19_. Notably, *C*_1_ represents the statistical data of daily rainfall during the rainy season in 2021. Conversely, qualitative indicators *C*_2_, and *C*_21_ to *C*_23_ were evaluated by five experts in the natural domain using heterogeneous data. Indicator *C*_20_ was assessed by four experts in the economic domain, while *C*_13_, *C*_16_, and *C*_17_ were evaluated by three experts in the social domain. The evaluation of all 23 indicators in 2021 is depicted in Table 5.

These qualitative evaluation data were subsequently converted into NCMs using the method described in Section 2.2, as shown in Table 6.

Finally, the 15 quantitative indicators were standardized and converted into NCMs through FPMF, as well as through the conversion of heterogeneous data. The eight qualitative indicators were transformed into NCMs using heterogeneous data conversion and the EGDMT, as illustrated in Table 7.

### 5.3. Comprehensive Evaluation and Analysis

In Section 5.1 and Section 5.2, the weights and evaluation results for the indicators are derived. Then, the evaluation results for Beijing’s flood resilience are computed using the weighted average cloud operator, as shown in Table 8.

As the cloud model incorporates *Ex*, *En*, and *He*, the assessment results are analyzed using a multi-area graph, taking into full consideration the uncertainty contained in the evaluation results. In the multi-area graph, the expected value curve is depicted by the central dark green line, demonstrating the variation of expected values with the indicator values. The regions transitioning from dark green to light green denote the “basic values”, “peripheral values”, and “uncertain values” areas in that order.

Consideration is given to the score cloud parameters (*Ex*, *En*, *He*). The “basic value” region corresponds to the score interval [*Ex* − *En*, *Ex* + *En*]. According to the characteristics of the normal cloud model, this interval contributes 68.26% of the total contribution. The “peripheral value” region includes the intervals [*Ex* − *En*, *Ex* − 3*En*] and [*Ex* + *En*, *Ex* + 3*En*], collectively accounting for 31.48% of the total contribution. Together, the “basic value” and “peripheral value” regions constitute 99.74% of the total contribution, indicating the fuzzy range of the score.

The outermost region, known as the “uncertain value” region, includes [*Ex* − 3*En*, *Ex* − 3(*En* + 3*He*)] and [*Ex* + 3*En*, *Ex* + 3(*En* + 3*He*)]. This area is subject to both subjective and objective uncertainty factors.

**Figure 4:**
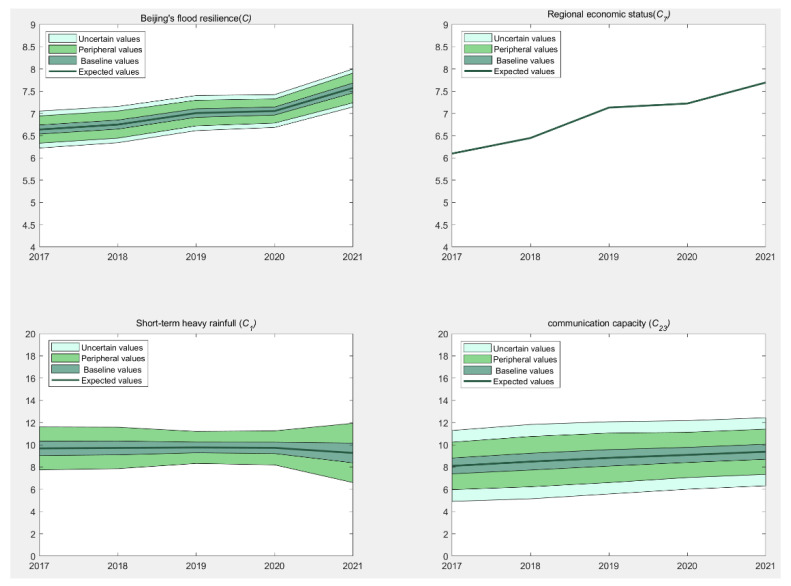
Changes in the evaluation results of Beijing’s urban flood resilience from 2017 to 2021 and the top three ranked indicators.

The evaluation results of Beijing’s urban flood resilience and the top three ranked indicators, regional economic status (*C*_7_), short-term heavy rainfall (*C*_1_), and communication capability (*C*_23_), from 2017 to 2021 are shown in Figure 4.

It can be seen from Figure 4. The change pattern of Beijing’s urban flood resilience can be summarized as a rapid increase during the periods of 2017–2019 and 2020–2021, with a slower increase between 2019 and 2020. The resilience score escalated from 6.6388 in 2017 to 7.5733 in 2021, reflecting a 14.1% enhancement. As shown in Figure 4 and Table 9, the rapid increase phase was primarily driven by significant improvements in the top ten indicators, including regional economic status (*C*_7_), communication capacity (*C*_23_), flood risk (*C*_2_), and drainability (*C*_9_). However, the slower increase phase occurred because the rate of improvement in these indicators decreased significantly, and the urban maintenance and construction budget (*C*_18_), ranked fourth, actually declined.

**Figure 5:**
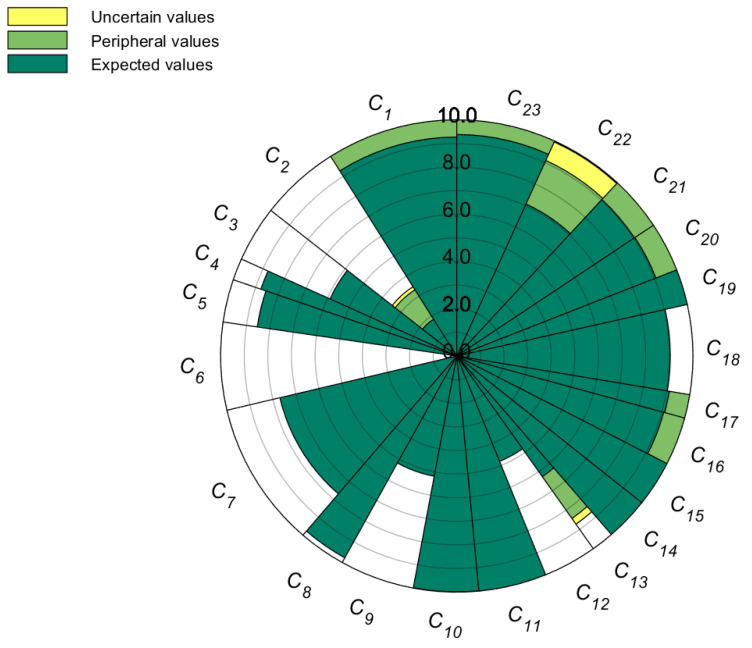
The evaluation results and weights of 23 indicators.

Figure 5 displays the weights of various indicators and their evaluation results for 2021. The angle of each sector illustrates the weight of the indicator. The green sector symbolizes the expected value of the indicator, while the light green region represents a portion of the “outer value” interval of the indicator, with a score range of [*Ex*, *Ex* + 3*En*]. The yellow sector signifies a segment of the “uncertain value” interval, characterized by a score range of [*Ex* + 3*En*, *Ex* + 3(*En* + 3*He*)].

When Figure 5 is correlated with Table 4, it becomes evident that among the top ten indicators ranked by weight, the scores of positive indicators *C*_7_, *C*_9_, and *C*_22_ necessitate enhancement. Such improvements could be realized through the development of the regional economy, expansion of drainage pipelines, and optimization of road traffic, thereby augmenting urban flood resilience in Beijing. On the other hand, the scores of negative indicators *C*_6_ and *C_2_* are relatively low, and increasing Beijing’s urban flood resilience by reducing flood risk and population density is necessary. High scores, such as *C*_23,_ *C*_18_, *C*_11_, and *C*_10_, pose greater challenges for improvement and can be maintained in their current state.

Moreover, the changes in uncertainty in the evaluation of Beijing’s urban flood resilience and major indicators are shown in Figure 5. Using the 2021 data as a reference, Table 10 illustrates the representative indicators *En*, 2*En*, and *He* for uncertainty values, outer values, and basic values, which are integral to the uncertainty representation of Beijing’s urban flood resilience. Figure 5 and Table 10 reveal that the uncertainty in Beijing’s urban flood resilience has significantly decreased compared to the uncertainty in major indicators. The decrease rate (DR) of Beijing’s urban flood resilience (*C*) is as high as 73.0% compared to that of the statistical indicator *C*_1_, and it is 64.6% compared to that of the qualitative indicator *C*_23_.

*C*–*C*_1_ represents the change in evaluation result *C* compared to the indicator *C*_1_ score, while *C*–*C*_23_ represents the change in evaluation result *C* compared to the indicator *C*_23_ score.

### 5.4. Sensitivity Analysis

λ denotes the adjustable parameter in the computation of indicator weights for the DM comprehensive weight, while μ is the adjustable parameter in the calculation of expert relative weights for indicator evaluation. Sensitivity analysis involves altering λ and μ from 0 to 1 to investigate the impacts of these adjustable parameters λ and μ on the final evaluation outcomes.

**Figure 6:**
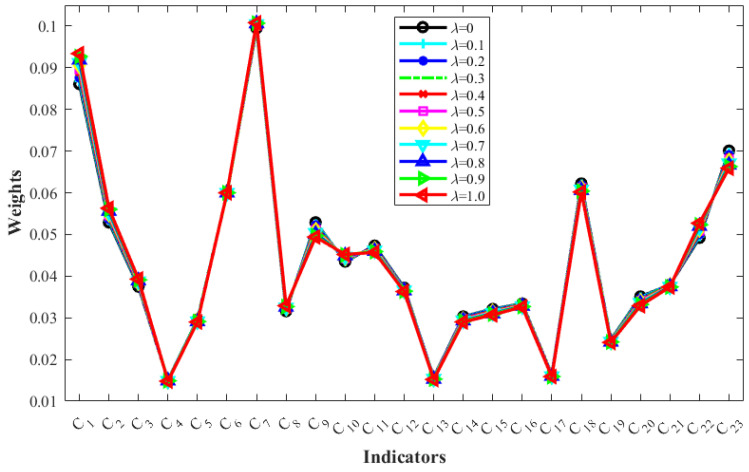
Changes in the indicator weight with λ.

The impact of the variation in the urban flood resilience indicator weights with respect to λ is shown in Figure 6. As depicted in this figure, with increasing λ, only slight variation occurs in the indicator weights. This outcome suggests that the weights of indicators are not sensitive to variations in λ, indicating a strong correlation between intrapersonal and interpersonal consistency in the computation of indicator weights. The employed method for calculating indicator weights, SW-GAHP, exhibits high robustness.

**Figure 7:**
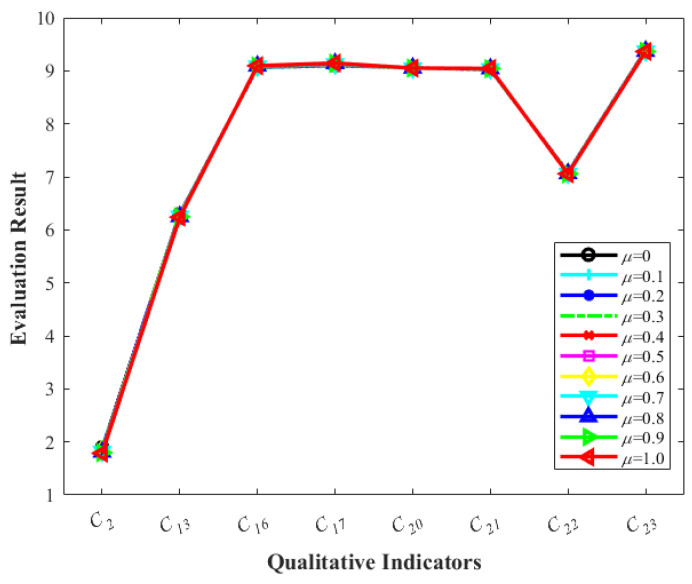
The indicator evaluation changes with μ.

The influence of varying μ on the qualitative evaluation outcomes of urban flood resilience indicators in 2021 is shown in Figure 7. During the evaluation of indicators, eight qualitative indicators were considered, originating from the social, economic, and natural domains. Table 11 displays the effect of *μ* on the DMs’ weights of three social domain indicators (*C*_13_, *C*_16_, and *C*_17_). This table indicates that the weights assigned by different experts to the same indicator exhibit notable variation with changes in *μ* values. However, as observed in Figure 7, the adjustable parameter μ exerts minimal influence on the evaluation results of the eight qualitative indicators, which remain largely constant. These findings suggest that the evaluation outcomes of qualitative indicators are not sensitive to μ, highlighting significant individual differences among experts but a negligible effect on collective results. This outcome also affirms the robustness of the EGDMT in indicator evaluation. 

**Figure 8:**
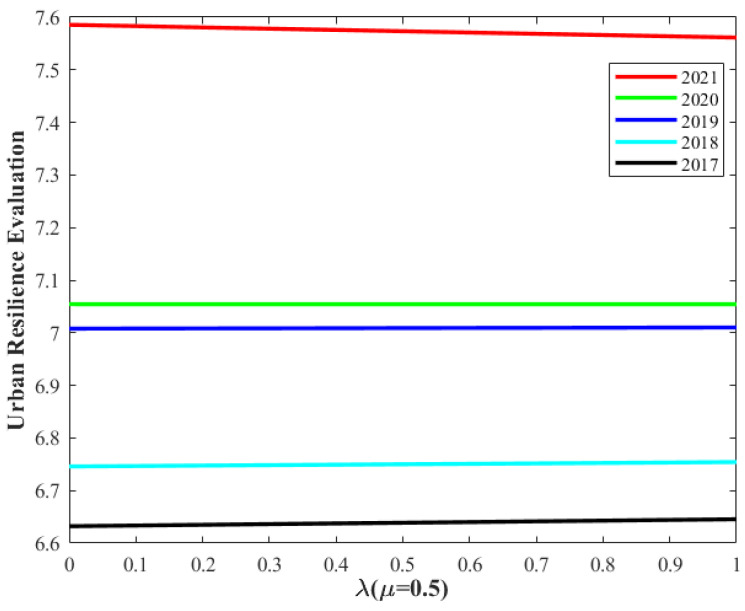
The urban flood resilience evaluation changes with λ.

**Figure 9:**
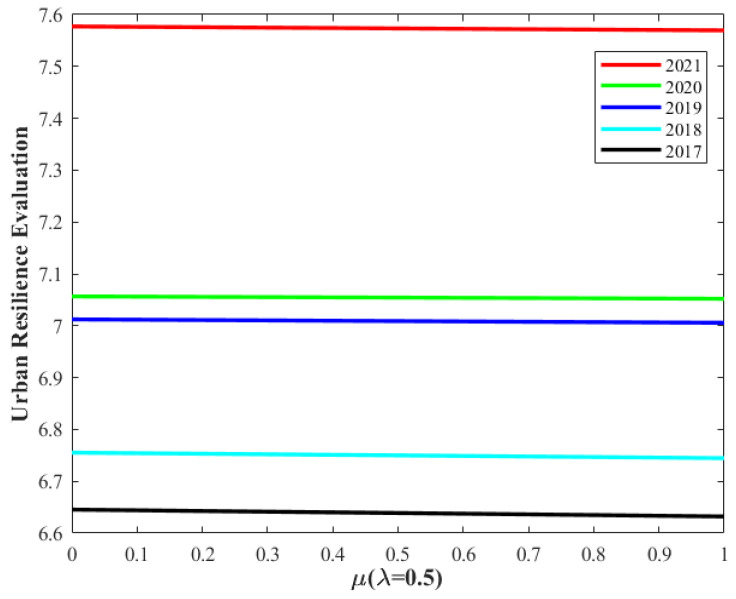
The urban flood resilience evaluation changes with μ.

Figure 8 and Figure 9 display the variations in the urban flood resilience evaluation results from 2017 to 2021. The analysis reveals that, on one hand, with *μ* = 0.5, irrespective of the *λ* value, the evaluation results from 2017 to 2021 exhibit only minor changes, while maintaining a consistent upward trend. On the other hand, with *λ* = 0.5, regardless of the *μ* value, the evaluation results for the same period remain nearly constant, with the overall upward trend continuing. These findings suggest that the urban flood resilience evaluation is not sensitive to either λ or μ, thus affirming the high robustness of the urban flood resilience assessment method proposed in this study. 

## 6. Discussion

A comprehensive evaluation model is proposed in this paper, including both quantitative and qualitative indicators. The SW-GAHP method effectively integrates the inputs of multiple decision-makers, assigning relevance to decision-makers based on their intrapersonal and interpersonal consistency. This approach aligns more closely with real-world scenarios, yielding more precise indicator weights. For the appraisal of qualitative indicators, the EGDMT, capable of handling heterogeneous data with various uncertainties, is proposed. The evaluation of quantitative indicators is facilitated through the use of FPMF. 

As far as we are aware, no research has been conducted that simultaneously takes into account heterogeneous data, qualitative and quantitative indicators, and an all-encompassing evaluation of urban flood resilience in the body of current literature. Since previous methods do not exhibit similar capabilities, direct computational comparisons are not feasible. Thus, this paper refrains from a direct methodological comparison. Instead, a comparative analysis is conducted to highlight the distinctive attributes of our proposed method. The comparison of our approach with several recent methodologies is detailed in Table 12.

Given the multitude of factors that must be considered in evaluating urban flood resilience, it is essential to devise a MCDM method that can effectively include both qualitative and quantitative indicators. Initially, in constructing the indicator systems, prior methodologies [11,15,61] employed their respective frameworks to thoroughly examine the disturbance process of urban floods. This analysis includes the “pressure” prior to the disturbance, the “state” during the disturbance, and the “adaptation” subsequent to the disturbance. However, there is a lack of comprehensive and systematic indicator screening methods. This paper uses a comprehensive SR indicator screening method and describes in detail the process of selecting 23 key indicators from 1172 related studies. This approach is more universal than the other methods and has certain guiding significance in this research field. Secondly, the resilience of quantitative indicators, when calculated using the linear normalization method, often fails to accurately represent the actual degree of resilience. In this paper, the resilience of quantitative indicators is determined through the FPMF method. This method is adept at adapting to both linear and nonlinear change rules of indicators with varying meanings, thus accurately reflecting their real impact on urban flood resilience. Thirdly, group decision-making technology is used to evaluate the resilience of qualitative indicators in the face of uncertainty. As indicated in Table 12, prior methods [11,15] are limited to handling exact numbers, and Li, Zhang, et al. [61] can also accommodate hesitant fuzzy information containing uncertainty. In contrast, the methodology proposed in this paper is capable of managing heterogeneous data, including exact numbers, statistical data, interval numbers, linguistic terms, and linguistic expressions. Regarding the allocation of indicator weights, previous methods [11,15,61] did not utilize group decision-making technology. While Zhang and Shang [61] involved five decision-makers in the evaluation of qualitative indicators, they only considered the group consensus among DMs for the weight of DMs. The SW-GAHP method allows multiple DMs to participate and assign the weight of indicators by combining the intrapersonal and interpersonal consistency of the DMs, effectively using collective intelligence. Finally, a novel qualitative indicators evaluation method, the EGDMT, is proposed. This method can handle and convey uncertain information during the evaluation process, and the final results incorporate both numerical values and uncertainties, a feature not found in previous methods [11,15,61], which lack the capability to manage uncertainty, yielding only deterministic results.

The application case and the comparative analysis herein demonstrate that this paper offers a more rational and effective approach for urban flood resilience evaluation. It utilizes a hybrid multi-indicator group decision-making method based on the PSR-SENCE model, heterogeneous data, and group decision-making techniques. The advantages of the methodology presented in this paper with regard to earlier approaches are clearly articulated.

(1). A comprehensive SR method was utilized to identify 23 critical influencing factors.
(2). A thorough qualitative and quantitative evaluation of urban flood resilience was conducted.
(3). The resilience evaluation of quantitative indicators accounted for both linearity and nonlinearity, in accordance with their respective meanings, employing the FPMF method for evaluation.
(4). Group decision-making technology is used to evaluate qualitative indicators, allowing multiple decision-makers in the field to participate while weighting based on the decision-maker’s contribution, considering intrapersonal consistency and interpersonal consistency.
(5). Heterogeneous data, including various uncertainties, such as exact numbers, statistical data, interval numbers, linguistic terms, NCMs, and linguistic expressions, are utilized to articulate evaluation results. This approach significantly increases the flexibility and applicability of knowledge representation across different stakeholders.
(6). A new method for determining indicator weights, designated SW-GAHP, is proposed. This method comprehensively assesses the decision-making quality in a rational manner. It ensures a more objective and precise determination of weights for each evaluation indicator.
(7). Cloud model theory is applied to manage the uncertainty inherent in heterogeneous data. The final result, represented by an NCM, incorporates not only numerical information but retains uncertainty information as well.
(8). A new evaluation method, EGDMT, is proposed to evaluate the qualitative indicators. This method facilitates a comprehensive and accurate evaluation of urban flood resilience without imposing additional burden on decision-makers, proving to be more reasonable and effective compared to previous methods.
(9). The hybrid MCGDM method is characterized by a clear problem description and a transparent implementation process. All calculations can be executed automatically through programming.

## 7. Conclusions

Extreme flooding is a significant challenge for urban development and building in an era of rapidly increasing population expansion and urbanization. This paper proposes a hybrid MCDM method based on the PSR-SENCE model, heterogeneous data, and group decision-making technology (GDMT). Based on the PSR-SENCE model, a combined indicator system including both quantitative and qualitative measures is constructed. Additionally, a new indicator weight calculation method called SW-GAHP is proposed. This method thoroughly considers both the intrapersonal consistency and interpersonal consistency of different DMs, increasing the rationality and accuracy of the weights of the indicators. An EGDMT method based on heterogeneous data is proposed for the evaluation of qualitative indicators. This method accounts for the uncertain degree and group consensus bias of DMs. It not only eases the burden on the super-decision-maker but also uses collective wisdom for decision-making, thereby minimizing the influence of individual biases on the overall evaluation. For the assessment of quantitative indicators, the more pragmatic FPMF method is utilized to enhance the rationality of quantitative indicator evaluation. Subsequently, the evaluation of both quantitative and qualitative indicators is converted into NCMs, enabling a consistent consideration of the uncertainty inherent in various heterogeneous data. The urban flood resilience evaluation not only includes numerical information but also retains uncertainty information, aligning more closely with human cognition and facilitating ease of understanding and acceptance. The evaluation of urban flood resilience in Beijing shows a year-on-year improvement from 2017 to 2021 with an overall increase of 14.1%. Moreover, based on the 2021 data, the uncertainty in the urban flood resilience evaluation of Beijing is significantly reduced, particularly when compared to the top three ranked indicators of short-term heavy rainfall and communication capacity. This underscores the effectiveness of the method that integrates qualitative and quantitative aspects, thereby enhancing the precision of urban flood resilience evaluation. Recommendations for positive indicators such as regional economic status, drainage capability, and public transportation service capacity include the vigorous development of regional economies, enhancement of urban drainage capacity, and optimization of urban road traffic management. For negative indicators like flood risk and population density, strategies involve reducing land population density through relevant diversion policies, enhancing residential environments, and decreasing the faced flood risk. These recommendations offer a theoretical foundation and decision-making guidance for improving urban flood resilience. Finally, through comparative and sensitivity analyses, the superiority and robustness of the method have been substantiated.

## Figures and Tables

**Table 1:** The meaning and evaluation method of each indicator.

Dimensions	Indicators	Description	Expression	Justification
Pressure				
Natural pressure	Short-term heavy rainfall (*C*_1_)	Daily rainfall statistics during the rainy season of the current year	$C_{1}=\{r_{d}^{1},r_{d}^{2},\ldots ,r_{d}^{n}\}$	[37,38,39]
	Flood risk (*C*_2_)	The degree of flood risk faced by the city	Fuzzy	[40,41,42]
	Topographic feature (*C*_3_)	Number between the highest and lowest points per area	$C_{3}=h_{h}-h_{l}$	[43]
State				
Social state	Special needs (*C*_4_)	Percentage of the population with disability	$C_{4}=\frac{\;N_{d}}{N_{tp}}$	[44,45,46]
	Vulnerable groups (*C*_5_)	Percentage of the population aged over 60 and under 15	$C_{5}=\frac{\;N_{uf}+N_{os}}{N_{tp}}$	[44,45,46]
	Population density (*C*_6_)	Population to area ratio	$C_{6}=\frac{\;N_{tp}}{S}$	[47]
Economic state	Regional economic status (*C*_7_)	Regional GDP data released by the National Bureau of Statistics	$C_{7}=V_{ragdp}$	[48,49]
	Unemployment rate (*C*_8_)	Proportion of urban unemployed population to total population	$C_{8}=\frac{\;N_{jp}}{N_{wp}}$	[50]
Natural state	Drainability (*C*_9_)	Density of urban drainage networks	$C_{9}=\frac{\;L_{tdn}}{Area}$	[51,52,53]
	Green coverage (*C*_10_)	Proportion of the vertical projection area of vegetation on the ground per area	$C_{10}=\frac{\;S_{g}}{S}$	[51,52,53]
	Density index of water network (*C*_11_)	Average amount of river distribution per area	$C_{11}=\frac{\sum_{i=1}^{m}N_{ri}}{m}$	[51,52,53]
Response				
Social response	Emergency services for response and recovery (*C*_12_)	Number of police stations, fire stations, and emergency operation centers per 10,000 population	$C_{12}=\frac{\sum_{i=1}^{3}N_{i}}{N_{tp}}$	[54,55,56]
	Citizen safety awareness and satisfaction (*C*_13_)	Citizens’ awareness of safety knowledge and their sense of safety attainment	Fuzzy	[57]
	Meteorological station network density (*C*_14_)	The number of regional meteorological stations	$C_{14}=\frac{N_{\mathrm{ms}}}{\;\mathrm{Area}}$	[4,58,59]
	Hydrological station network density (*C*_15_)	The number of regional hydrological stations	$C_{15}=\frac{N_{\mathrm{hgs}}}{\;\mathrm{Area}}$	[4,58,60]
	Early warning capacity (*C*_16_)	Comprehensive prediction accuracy and warning efficiency	Fuzzy	[4,58,61]
	Knowledge learning ability (*C*_17_)	The ability to learn from and simulate past disaster experiences	Fuzzy	[62]
Economic response	Urban maintenance and construction budget (*C*_18_)	The proportion of financial expenditure dedicated to maintaining public safety in the city	$C_{18}=\frac{\;E_{psf}}{E_{clf}}$	[63]
	Flood insurance (*C*_19_)	Per capita insurance costs in the city	$C_{19}=\frac{\;E_{fir}}{N_{tp}}$	[64,65]
	Economic diversity (*C*_20_)	The richness or diversity of the city’s economic forms	Fuzzy	[66]
Natural response	Food availability (*C*_21_)	The food supply capacity for the affected population in the city	Fuzzy	[67,68,69]
	Public transportation service capacity (*C*_22_)	The transportation capacity for personnel and materials	Fuzzy	[42,67,68]
	Communication capacity (*C*_23_)	The communication capability for emergency rescue operations	Fuzzy	[67,68,70]

**Table 2:** FPMFs and relevant parameters for different indicators.

Indicators	*x*_min_	*x*_max_	*k*	FPMF
*C*_1_	0	250	2	CvDF
*C*_3_	200	2000	1	CvDF
*C*_4_	0	5%	3	CcDF
*C*_5_	0	50%	3	CcDF
*C*_6_	0	100	2	CcDF
*C*_7_	0.6607	58.412	4	CcIF
*C*_8_	2%	10%	2	CcDF
*C*_9_	0	3.76	2	CcIF
*C*_10_	25%	50%	2	CcIF
*C*_11_	0	2269	3	CcDF
*C*_12_	0	1	2	CcIF
*C*_14_	0	500	2	CvIF
*C*_15_	100	20,000	2	CcDF
*C*_18_	0	10%	2	CcIF
*C*_19_	0	10,000	2	CcIF

**Table 3:** The first level indicators’ six PCMs from six DMs.

1				1				1				1	2	1		1				1		
2	1			3	1	1		3	1	1			1			4	1	2		3	1	
4	2	1		3		1		3		1			2	1		2		1		3	2	1

**Table 4:** The weight calculation results for the indicators from 2017 to 2021.

	***C*_1_**	***C*_2_**	***C*_3_**	***C*_4_**	***C*_5_**	***C*_6_**	***C*_7_**	***C*_8_**	***C*_9_**	***C*_10_**	***C*_11_**	***C*_12_**
Weight	0.0897	0.0546	0.0384	0.0150	0.0293	0.0599	0.1002	0.0323	0.0511	0.0444	0.0465	0.0367
Rank	2	6	11	23	19	5	1	16	7	10	9	13
	***C*_13_**	***C*_14_**	***C*_15_**	***C*_16_**	***C*_17_**	***C*_18_**	***C*_19_**	***C*_20_**	***C*_21_**	***C*_22_**	***C*_23_**	
Weight	0.0155	0.0297	0.0314	0.0330	0.0161	0.0612	0.0244	0.0340	0.0376	0.0510	0.0680	
Rank	22	18	17	15	21	4	20	14	12	8	3	

**Table 5:** The evaluation of 23 indicators in 2021.

	*C*_1_	*C*_3_	*C*_4_	*C*_5_	*C*_6_	*C*_7_	*C*_8_	*C*_9_
*2021*	--	940.71	2.49%	26.34%	1334	18.398	3.20%	1.15
	***C*_10_**	***C*_11_**	***C*_12_**	***C*_14_**	***C*_15_**	***C*_18_**	***C*_19_**	
	49%	166	0.25	514	37.17	6.90%	3795.51	
	***C*_2_**		***C*_21_**		***C*_22_**		***C*_23_**	
	*DM*_1_: *[1, 2]* *DM*_2_: *low* *DM*_3_: (1.5, 0.5, 0.033) *DM*_4_: *lower than low* *DM*_5_: 1.5		*DM*_1_: *[8, 9]* *DM*_2_: *high* *DM*_3_: (8.5, 0.5, 0.033) *DM*_4_: *greater than high* *DM*_5_: 8.3		*DM*_1_: *[7, 8]* *DM*_2_: *sh* *DM*_3_: (7.5, 0.5, 0.033) *DM*_4_: *between sh and high* *DM*_5_: 7.5		*DM*_1_: *[8.5, 9.5]* *DM*_2_: *vh* *DM*_3_: (9.5, 0.5, 0.033) *DM*_4_: *between high and vh* *DM*_5_: 9.4	
	***C*_13_**		***C*_16_**		***C*_17_**		***C*_20_**	
	*DM*_6_: 7 *DM*_7_: *sh* *DM*_8_: between *sh* and *high*		*DM*_6_: *8.8* *DM*_7_: *high* *DM*_8_: between *high* and *vh*		*DM*_6_: *8* *DM*_7_: *sh* *DM*_8_: between *sh* and *high*		*DM*_9_: *8.2* *DM*_10_: (8.3, 0.5, 0.023) *DM*_11_: *[8, 8.6]* *DM*_12_: *lower than high*	

**Table 6:** The NCMs for 5 years according to the 23 indicators.

	*C*_2_	*C*_21_	*C*_22_	*C*_23_
*2021*	*DM*_1_: (1.5000, 0.1667, 0) *DM*_2_: (1.2460, 1.0620, 0.0340) *DM*_3_: (1.5000, 0.5000, 0.0330) *DM*_4_: (0.0985, 0.4570, 0.0297) *DM*_5_: (1.5000, 0, 0)	*DM*_1_: (8.5000, 0.1667, 0) *DM*_2_: (8.7430, 1.2420, 0.0410) *DM*_3_: (8.5000, 0.5000, 0.3300) *DM*_4_: (9.8875, 1.0540, 0.0336) *DM*_5_: (8.3000, 0, 0)	*DM*_1_: (7.5000, 0.1667, 0) *DM*_2_: (7.1050, 1.0270, 0.0410) *DM*_3_: (7.5000, 0.5000, 0.3300) *DM*_4_: (7.9240, 1.4075, 0.0580) *DM*_5_: (7.5000, 0, 0)	*DM*_1_: (9.0000, 0.1667, 0) *DM*_2_: (9.7750, 1.0540, 0.0270) *DM*_3_: (9.5000, 0.5000, 0.3300) *DM*_4_: (9.2590, 1.3200, 0.0491) *DM*_5_: (9.4000, 0, 0)
	***C*_13_**	***C*_16_**	***C*_17_**	***C*_20_**
	*DM*_1_: (7.0, 0, 0) *DM*_2_: (7.1050, 1.0270, 0.0410) *DM*_3_: (7.9240, 1.4075, 0.0580)	*DM*_1_: (8.80, 0, 0) *DM*_2_: (8.7430, 1.2420, 0.0410) *DM*_3_: (9.2590, 1.3200, 0.0491)	*DM*_1_: (8.0, 0, 0) *DM*_2_: (7.1050, 1.0270, 0.0410) *DM*_3_: (7.9240, 1.4075, 0.0580)	*DM*_1_: (8.2, 0, 0) *DM*_2_: (8.3, 0.5000, 0.0230) *DM*_3_: (8.3, 0.1, 0) *DM*_4_: (7.9240, 1.4075, 0.0580)

**Table 7:** The NCMs of 23 indicators in 2021.

	*C*_1_	*C*_2_	*C*_3_	*C*_4_	*C*_5_
*2021*	(9.2698, 0.8889, 0)	(1.8401, 0.4911, 0.0172)	(5.8849, 0, 0)	(8.7649, 0, 0)	(8.5380, 0, 0)
	***C*_6_**	***C*_7_**	***C*_8_**	***C*_9_**	***C*_10_**
	(0, 0, 0)	(7.6954, 0, 0)	(9.7750, 0, 0)	(5.1816, 0, 0)	(9.9840, 0, 0)
	***C*_11_**	***C*_12_**	***C*_13_**	***C*_14_**	***C*_15_**
	(9.9961, 0, 0)	(4.8160, 0, 0)	(6.2672, 0.7222, 0.0328)	(10, 0, 0)	(9.9999, 0, 0)
	***C*_16_**	***C*_17_**	***C*_18_**	***C*_19_**	***C*_20_**
	(9.0809, 0.7731, 0.0272)	(9.1224, 0.7755, 0.0273)	(9.0390, 0, 0)	(10, 0, 0)	(9.0549, 0.5483, 0.0731)
	***C*_21_**	***C*_22_**	***C*_23_**		
	(9.0333, 0.6885, 0.1034)	(7.0694, 0.6756, 0.0959)	(9.3771, 0.6774, 0.1143)		

**Table 8:** The evaluation results for Beijing’s flood resilience from 2017 to 2021.

	2021	2020	2019	2018	2017
Result	(7.5733, 0.1111, 0.0104)	(7.0543, 0.0902, 0.0109)	(7.0090, 0.0962, 0.0118)	(6.7500, 0.1016, 0.0114)	(6.6388, 0.1021, 0.0121)

**Table 9:** Evaluation results of key indicators of Beijing’s urban flood resilience from 2017 to 2021.

	2017	2018	2019	2020	2021
*C*_18_	(9.0140, 0, 0)	(9.0452, 0, 0)	(9.2710, 0, 0)	(9.1590, 0, 0)	(9.0390, 0, 0)
*C*_2_	(1.6160, 0.6740, 0.0849)	(1.7054, 0.6730, 0.0686)	(1.7738, 0.6778, 0.1011)	(1.6671, 0.4989, 0.0613)	(1.8401, 0.4911, 0.0172)
*C*_9_	(4.6896, 0, 0)	(4.8817, 0, 0)	(4.9575, 0, 0)	(4.9575, 0, 0)	(5.1816, 0, 0)

**Table 10:** The uncertainty of the evaluation of urban flood resilience and major indicators.

	*En*	2*En*	*He*	DR/%
*C*	0.2399	0.4798	0.0104	-
*C*_7_	0	0	0	-
*C*_1_	0.8889	1.7778	0	-
*C*_23_	0.6774	1.3548	0.1143	-
*C*–*C*_1_	-	-	-	73.0
*C*–*C*_23_	-	-	-	64.6

**Table 11:** Variation in the weights assigned by DM in the social domain with respect to μ.

μ	*C*_13_			*C*_16_			*C*_17_		
	$\chi^{1}$	$\chi^{2}$	$\chi^{3}$	$\chi^{1}$	$\chi^{2}$	$\chi^{3}$	$\chi^{1}$	$\chi^{2}$	$\chi^{3}$
0.0	0.3157	0.2951	0.3893	0.2933	0.3405	0.3663	0.2899	0.3334	0.3767
0.5	0.4078	0.2936	0.2985	0.3966	0.2998	0.3036	0.395	0.2962	0.3088
1.0	0.5	0.2922	0.2078	0.5	0.259	0.241	0.5	0.259	0.241

**Table 12:** Analytical comparison using recent methods.

Methods	Indicator Selection	Indicator Evaluations	Data Types	Indicator Weights	Expert Weights	Result
Zhang, Shang. [11]	PSR-SEEI	Linear normalization	Exact numbers	EWM+AHP	--	a crisp number
Zhang et al. [15]	SEIE	Linear normalization	Exact numbers	AHP	--	a crisp number
Li, Zhang, et al. [61]	RCRA	Quantitative indicators: linear normalization Qualitative indicators: Group decision-making	Exact numbers Hesitant fuzzy set	weighted averaging operator	minimum divergence model	a crisp number
Proposed method	PSR-SENCE+SR	Quantitative indicators: FPMF Qualitative indicators: Group decision-making	Exact numbers Interval numbers Statistical numbers Linguistic terms Linguistic expressions	SW-GAHP	SWM UD+GB	an NCM

## Data Availability

The datasets generated during and/or analyzed during the current study are available from the corresponding author on reasonable request.
